# Aneurism of Thoracic Aorta

**Published:** 1855-09

**Authors:** 


Aneurism of Thoracic Aorta.—1. That there are no peculiar or consistent signs belonging to aneurism, by which the sounds produced in the tumor can be distinguished from those of the heart.

2. That, as a general rule, the sounds of an aneurism are more and more similar to those of the heart according as the seat of disease approaches the origin of the aorta.

3. That the statement of Laennec, as to the singleness of the aneurismal sound, and that of Hope, who maintains the existence of unequivocal criteria, distinguishing the aneurismal from the cardiac sounds, cannot be admitted.

4. That murmur is frequently absent in aneurisms of the arch of the aorta and the innominata.

5. That the discovery of two centers of pulsation within the thorax, as indicated by impulse and by a single or by double sounds, is the simplest expressions of the physical diagnosis.

6. That an extremely weak, almost imperceptible, impulse may attend even a large aneuism of the aorta.

7. That the disease cannot always be detected by percussion.

8. That the double sounds are met with in the true as well as the false aneurisms of the aorta.

9. That the first aneurismal sound is not necessarily, as Hope teaches, a murmur.

10. That we cannot yet explain the presence or absence of murmur in many cases of aneurism.

11. That in certain cases the diagnosis of a communicating aneurism may be ventured on, especially where the signs as indicated by Mr. Thurnam have occurred within a short space of time; and with still more certainty if they have supervened upon some violent effort.

12. That the signs from compression of surrounding parts may be arranged according to their importance as follows:

*a*. Pressure on the trachea and bronchial tubes.

*b*. Compression and sometimes obliteration of blood-vessels.

*c*. Pressure on the œsophagus.

*d*. Pressure on the nerves.

13. That the amount of pressure varies within short periods of time.

14. That in consequence of a change in the direction of pressure, parts whose functions had been interrupted or injured may be perfectly relieved.

15. That these circumstances may assist in the diagnosis between cancerous aneurismal tumors.

16. That in certain cases of aneurismal pressure, the effects on the parts engaged has probably a double origin; on the one hand resulting from mechanical, and on the other from vital causes.

17. That aneurismal may often be distinguished from laryngeal stridor by observing the source and direction of the sound.

18. That a small aneurism, causing lateral compression of the trachea, will sooner produce stridor than a larger tumor, the pressure of which is more directly from before backward.

19. That a considerable narrowing of the tube has been produced, yet without stridor.

20. That a degree of the stridor from below may exist, even though the trachea be not compressed, if the tumor engages one of the primary divisions of the tube.

21. That in many cases inequality of vesicular murmur, unattended by signs or symptoms of pulmonary disease, is produced by the compression of one of the bronchial tubes.

22. That this diminution of the vesicular murmur is generally equable over the affected side, but may be confined to the upper portion of the lung.

23. That in certain cases absence of murmur during the first half of the respiratory effort may be observed.

24. That in addition we may enumerate, among the consequences of this pressure on one bronchial tube, the unequal expansion of the sides during respiration; the want of vocal vibration to the hand; and lastly, as Dr. Mayne has observed, the actual contraction of one side, similar to that produced after the absorption of an empyema.

25. That in these cases we may have stridor combined with alteration or loss of voice; lesion of voice without stridor; and, lastly, stridor without any change of voice.

26. That aphonia, strictly speaking, is rarely observed in aneurism; the voice may be variously altered [metaphonia], but is seldom wholly extinct.

27. That variation of the voice, within short periods of time, is often characteristic of aneurismal pressure.

28. That in the course of the disease, the signs of pressure on the arteries may disappear, and the pulse at the distal side of the tumor be fully restored.

29. That in certain cases permanent obliteration of arterial canals is produced, and in this way important organs, such as the brain and lungs, may suffer from the want of arterial supply.

30. That aneurismal dysphagia may occur without stridor or signs of compression of the arteries or veins.

31. That it may exist to a great degree, and yet the passage of a probang be effected without difficulty.

32. That it may be referred to different portions of the tube within spaces of time.

33. That in a case where there was evidence of the retreat of the aneurismal tumor from the front of the thorax, and in which great destruction of several vertebræ had taken place, the signs of compression were observed to reappear on the removal of mechanical support to the shoulders.

34. That the force of the aneurismal diastole cannot be taken as a measure of that of the heart.

35. That in certain cases in which two centers of pulsation are discoverable, the feebleness of the cardiac, as compared with the aneurismal impulse, greatly facilitates the diagnosis.

36. That the existence even of a large aneurism of the thoracic aorta seems to have little if any influence in producing hypertrophy of the heart.

37. That atrophy of the heart may co-exist with a large aneurism; and that the frequent absence of cardiac disease in aneurism facilitates the diagnosis of the latter disease, more especially where the pulsations of the aneurism are attended with murmur.

38. That of the general morbid conditions which accompany aneurism, tubercular phthisis is the most common.

39. That perforation of hollow organs may take place without the patient dying of hemorrhage.

40. That even in cases of external opening there may be many successive hemorrhages, or the blood may ooze out, or be discharged in a small continuous stream.

41. That when the aneurism opens into a free serous cavity, the first gush of blood is not necessarily fatal, nor even the second.

42. That where it opens into a serous cavity in which partial adhesions exist, death may be gradual.

43. That sudden death may occur in aneurism without any rupture of the sac.

44. That when the communication is formed between the sac and the vena cava, or the right auricle, the symptoms of venous congestion are produced.—*Stokes' Diseases of the Heart & Aorta.*

